# Effect Modification by Attention Deficit Hyperactivity Disorder (ADHD) Symptoms on the Association of Psychosocial Work Environments With Psychological Distress and Work Engagement

**DOI:** 10.3389/fpsyt.2019.00166

**Published:** 2019-03-27

**Authors:** Masako Nagata, Tomohisa Nagata, Akiomi Inoue, Koji Mori, Shinya Matsuda

**Affiliations:** ^1^Department of Occupational Health Practice and Management, Institute of Industrial Ecological Sciences, University of Occupational and Environmental Health, Kitakyushu, Japan; ^2^Data Science Center for Occupational Health, University of Occupational and Environmental Health, Kitakyushu, Japan; ^3^Department of Public Health, Kitasato University School of Medicine, Sagamihara, Japan; ^4^Department of Preventive Medicine and Community Health, School of Medicine, University of Occupational and Environmental Health, Kitakyushu, Japan

**Keywords:** ADHD, job demands, job control, social support, psychological distress, work engagement

## Abstract

**Objective:** The aim of this study was to examine how attention-deficit hyperactivity disorder (ADHD) symptoms play an interaction effect on the association between psychosocial work environments and health (psychological distress/work engagement) among workers.

**Methods:** This is a cross-sectional study of 2,693 employees at a pharmaceutical company using a self-administered questionnaire evaluating ADHD symptoms (Adult ADHD Self-Report Scale Screener), psychosocial work environments (job demands, job control and social support), and health outcomes (psychological distress; K6, and work engagement; Utrecht Work Engagement Scale). Multiple regression analyses were applied to assess the interaction between ADHD symptoms and psychosocial work environments on health outcomes.

**Results:** The prevalence of workers with ADHD symptoms was 5.9% (n = 159). Significant interaction effects of ADHD symptoms × job control and ADHD symptoms × social support were observed (β = −0.067, *p* < 0.01 and β = −0.052, *p* < 0.01, respectively) on psychological distress after adjustment of age, sex, occupation and education. The interaction effect of ADHD symptoms × each psychosocial work environment was not observed on work engagement.

**Conclusions:** Job control and social support were more influential factors that were related to psychological distress in accordance with ADHD symptoms. This study also found no difference of the interaction between psychosocial work environments and ADHD symptoms on work engagement. To the best of our knowledge, this study was first to clarify the effect of ADHD symptoms on the association between psychosocial work environments and health outcomes (psychological distress/work engagement). These findings can aid employers how to arrange better work environments for workers with ADHD symptoms.

## Introduction

Attention-deficit hyperactivity disorder (ADHD) is a neuropsychiatric disorder characterized by inattention, excessive motor activity, and impulsivity. ADHD starts in childhood and can persist into adolescence and adulthood (1, 2). Adults with ADHD experience difficulties in social interactions and are more likely to suffer from depression and social anxiety disorder (2). Workers with ADHD tend to show work impairment and reduced productivity (e.g., absenteeism and presenteeism) (3). Furthermore, adults with ADHD are at increased risk of accidents, trauma, and workplace injuries (4).

Individuals with ADHD are common in the workplace. An estimated 1.9–4.2% of workers are reported to have adult ADHD in the United States, and an average of 3.5% was found among workers in 10 countries (i.e., Belgium, Colombia, France, Germany, Italy, Lebanon, Mexico, the Netherlands, Spain, and the United States) (5–7). Moreover, it is known that ADHD symptoms exist on a continuum, and therefore there are people with subthreshold ADHD. Similar to adults with ADHD, those with subthreshold ADHD are also at risk of adverse outcomes (8, 9). It is likely that some workers with mental disorders have undiagnosed ADHD or subthreshold ADHD (10, 11), and these individuals with ADHD symptoms may be at a higher risk for developing a mental health disorder.

The mental health of workers is associated with the psychosocial work environments. The past study indicated that unfavorable psychosocial work environments, such as high job demands, low job control and low social support are associated with psychological distress of workers (12). It was known that high job demands and low job control are prospective risk factors for common mental disorders (12, 13). Psychosocial work environments affect not only poor health, but also positive aspects of health such as work engagement. Work engagement is of strong interest to employers because lower work engagement leads to greater loss of productivity (14). High job control and high social support are intrinsically and/or extrinsically motivating and therefore taken as the primary drivers of work engagement (15–18). Adults with ADHD symptoms such as hyperactivity-impulsivity and inattention (1) tend to experience problems with working memory, planning, and anticipation (19, 20). These deficits are closely linked to the ability to function at work (21–23). The past qualitative study suggested that coping with workload is a key point for employed women with ADHD to success in employment (24). This suggests that psychosocial work environments are more important key factors to fit to work for workers with ADHD symptoms. To the best of our knowledge, however, it is not clear what role ADHD symptoms would play in the relationships between psychosocial work environments and negative/positive well-being as the health outcome.

The purpose of this study was to investigate how ADHD symptoms modify the relationship psychosocial work environments and negative/positive aspects of mental health (psychological distress/work engagement) for workers.

We have two hypotheses.

Hypothesis 1. ADHD symptoms enhance the association between low job control and psychological distress.

Workers with ADHD symptoms have the difficulty to follow the instructions of others (25), and then job control might be more important factor to fit to work among workers with ADHD symptoms.

Hypothesis 2. ADHD symptoms enhance the association between high job control and work engagement.

Adults with ADHD tend to be resilient and creative, to have foresight, and to generate ideas (26). Workers with ADHD often have the entrepreneurship and professionalism (27). ADHD may offer performance advantages in entrepreneurial environments or in professions where rapid decision making or creativity is required (26–28). Job control is high in these professions. That is why the association between high job control and work engagement may be stronger among workers with ADHD symptoms.

In addition to two hypotheses, we conducted the exploratory analyses to investigate how ADHD symptoms modify the relationship other psychosocial work environments (job demands and social support) and health for workers.

## Methods

### Participants

We distributed self-administered questionnaires to employees of a pharmaceutical company. Participation was voluntary. Company health care staff sent an email in September 2016 to all employees (*n* = 4,738; male = 3,361, female = 1,377) to inform them about the study and encourage them to complete the questionnaire. The questionnaire included psychosocial work environments, psychological distress, work engagement, and the Adult ADHD Self-Report Scale short version (ASRS screener) (29). Only the researchers had access to results of the questionnaire (the company staff did not).

The research protocol was approved by the Ethics Committee of Medical Research, University of Occupational and Environmental Health, Japan.

### Measures

#### ADHD

The short-form ASRS Screener is available in many languages and comprises a checklist of six questions about ADHD symptoms based on the diagnostic criteria of the Diagnostic and Statistical Manual of Mental Disorders, Fourth Edition, Text Revision (30). Respondents rate the frequency with which individual ADHD symptoms have occurred over the past 6 months using a five-point Likert scale (0 = never, 1 = rarely, 2 = sometimes, 3 = often, and 4 = very often). Response scores are equally weighted and summed to generate a total score ranging from 0 to 24, with higher scores indicating an increased risk of ADHD. Cronbach's alpha coefficient was 0.61 for this sample. To screen for ADHD, dichotomous responses to each of the six questions were counted; respondents who made a positive response in 4 or more questions were considered to have a positive screening (29), and were placed in the workers with ADHD symptoms. To examine the interaction between the severity of ADHD symptoms and psychosocial work environments, total ASRS score was used.

#### Psychosocial Work Environments

We used the Brief Job Stress Questionnaire (BJSQ) to measure job stressors (31). The BJSQ is a standardized instrument used to assess social and psychological characteristics of jobs based on the job stress model developed by the group of researchers from the US National Institute for Occupational Safety and Health (NIOSH) (31). We assessed three work components in the BJSQ: job demands (six items), job control (three items), and social support at the workplace (six items). There are four response options to each question, ranging from strongly agree to strongly disagree.

Job demands include qualitative job overload (three items) and quantitative job overload (three items). Total possible score ranges from 6 to 24. Job control consists of three items, and the total possible score for job control ranges from 3 to 12. Social support at the workplace includes supervisor support (three items) and coworker support (three items), with total possible scores ranging from 6 to 24. Cronbach's alpha coefficient of job demands, job controls, and social support were 0.79, 0.83, and 0.88, respectively.

#### Outcome: Psychological Distress

Psychological distress was measured using the Japanese version of the K6 scale, which has demonstrated acceptable internal consistency, reliability, and validity (32). The K6 scale comprises six items measuring psychological distress level on a five-point scale ranging from 0 (none of the time) to 4 (all of the time) (total score range: 0–24). Cronbach's alpha coefficient for this sample was 0.89.

#### Outcome: Work Engagement

Work engagement was assessed using the nine-item Japanese version of the Utrecht Work Engagement Scale (UWES-9) (33). The UWES-9 was developed in order to measure the characteristics of vigor, dedication, and absorption. Items are rated on a seven-point response scale, from 0 (never) to 6 (always), resulting in a total score ranging from 0 to 54. The Japanese translation of the UWES-9 has demonstrated acceptable internal consistency reliability, as well as factor and construct validity (34). Cronbach's alpha coefficient for this sample was 0.95.

#### Demographic Characteristics

The questionnaire also measured the following demographic characteristics: sex, age, education, and occupation. Age was used as a continuous variable. Education was categorized into four groups (less than 12 years, 12–14 years, 16–18 years, and more than 18 years). Occupation was categorized into six groups based on the categories established in the International Standard Classification of Occupation: supervisory, clerical, blue collar, sales, research, and technical. Dummy variables were created using the clerical category as a reference.

### Statistical Analysis

We calculated means and standard deviations, or proportion of the demographic characteristics of participants. To assess for interaction between the severity of ADHD symptoms and psychosocial work environments, we conducted multiple regression analyses using ASRS totals as continuous variables among all participants. Prior to testing, the total ASRS, job demands, job control, and social support scores were mean-centered. ASRS scores, job demands × ASRS, job control × ASRS, and social support × ASRS were included as independent variables. Psychological distress and work engagement were used as dependent variables. We first conducted a crude model (Model 1), then adjusted for age and sex to generate a second model (Model 2). Psychosocial work environments differ depending on occupation, and health outcomes differ depending on socioeconomic status such as education. To confirm the consistency of results, we created additional models adjusted for occupation (Model 3), and education (Model 4). When significant interaction effects of psychosocial work environments × ASRS were observed, we conducted *post-hoc* simple slope analyses at one standard deviation above/below the mean score of ASRS scores. In a series of analyses, R^2^, adjusted R^2^, and ΔR^2^ were calculated in each step to assess model fit. In addition, residual analyses were conducted to estimate the amount of autocorrelation in the residuals using the Durbin-Watson statistic (ranging from 0 to 4.0 and a value of 2.0 means that there is no autocorrelation). The level of significance was 0.05. Statistical analyses were performed using IBM SPSS Statistics for Windows, Version 24 (IBM Corporation, Armonk, NY).

## Results

The respondents were 2,791 workers out of all employees (response rate = 59.8%). The proportion of women and mean age of respondents and all employees were 28.1%, 42.7 and 29.1%, 42.5, respectively. After excluding cases with missing data, 2,693 cases were analyzed as complete cases. In this sample, the prevalence of workers with ADHD symptoms was 5.9% (*n* = 159). Table 1 shows the demographic characteristics (sex, age, education, and occupation) and the means and standard deviations of psychosocial work environments, ASRS, and health outcomes (psychological distress and work engagement) scores of participants in this study.

**Table 1:** Demographic characteristics of participants.

	**Mean (SD)**	***n* (%)**
**SEX**		
Male		1,943 (72.2)
Female		750 (27.8)
**AGE**		
	42.8 (9.8)	
**EDUCATION**		
−12 years		346 (12.8)
12–14 years		255 (9.5)
16–18 years		1,061 (39.4)
18- years		689 (25.6)
No response		342 (12.7)
**OCCUPATION**		
Supervisory		759 (28.2)
Clerk		441 (16.4)
Blue collar		268 (10.0)
Sales		622 (23.1)
Research		545 (20.2)
Technical		548 (2.2)
**PSYCHOSOCIAL WORK ENVIRONMENTS**		
Job control	9.0 (1.7)	
Job demands	18.4 (3.1)	
Social support	18.7 (3.5)	
ASRS scores	13.1 (3.2)	
**HEALTH OUTCOME**		
Psychological distress^†^	2.9 (3.7)	
Work engagement^‡^	29.2 (9.2)	

† *Psychological distress was evaluated by K6 scale*.

‡ *Work engagement was evaluated by Utrecht Work Engagement Scale*.

Table 2 shows the main effects and interaction effects of psychosocial work environments and ADHD symptoms on psychological distress among all participants. After adjusting for demographic characteristics (Model 4), a significant interaction effect of job control × ASRS (ADHD symptoms) on psychological distress was observed (β = −0.067, *p* < 0.01), as well as a significant interaction effect of social support × ASRS on psychological distress (β = −0.052, *p* < 0.01). However, no significant interaction of job demands × ASRS on psychological distress was seen. Because we observed significant interactions of job control × ASRS, and social support × ASRS, we conducted *post-hoc* simple slope analyses at one standard deviation above/below the mean ASRS score. The *post-hoc* simple slope analyses showed that the simple slopes of job control and social support were greater at higher levels of ASRS compared to the lower levels of ASRS.

**Table 2:** Main effects and interaction effects of ASRS and job control, job demands, and social support on K6 values, and the simple slope of ASRS according to level of job control and social support on psychological distress (K6) among all participants.

	**Model 1^a^**	**Model 2^b^**	**Model 3^c^**	**Model 4^d^**
	**β**	**β**	**β**	**β**
ASRS	0.359^*^	0.346^*^	0.345^*^	0.346^*^
**Psychosocial work environments**				
Job control	−0.144^*^	−0.140^*^	−0.136^*^	−0.134^*^
Job demands	0.102^*^	0.111^*^	0.115^*^	0.113^*^
Social support	−0.230^*^	−0.231^*^	−0.229^*^	−0.230^*^
**Psychosocial work environments** **×** **ASRS (ADHD symptoms)**				
Job control × ASRS (ADHD symptoms)	−0.063^*^	−0.068^*^	−0.067^*^	−0.067^*^
Job demands × ASRS (ADHD symptoms)	0.004	0.005	0.005	0.005
Social support × ASRS (ADHD symptoms)	−0.050^*^	−0.051^*^	−0.052^*^	−0.052^*^
*R*^2^	0.291	0.301	0.301	0.301
Adjusted *R*^2^	0.289	0.298	0.298	0.298
Δ*R*^2^	0.291^*^	0.009^*^	0.001	0.001
Durbin-Watson				2.041
**Simple slope (job control)**				
ASRS high score group (One SD above the mean)	−0.202^*^	−0.203^*^	−0.200^*^	−0.196^*^
ASRS low score group (One SD below the mean)	−0.088^*^	−0.081^*^	−0.079^*^	−0.076^*^
**Simple slope (social support)**				
ASRS high score group (One SD above the mean)	−0.275^*^	−0.278^*^	−0.280^*^	−0.279^*^
ASRS low score group (One SD below the mean)	−0.184^*^	−0.183^*^	−0.185^*^	−0.183^*^

*Psychological distress was evaluated by K6 scale*.

*β: Standardized coefficient (β)*.

a *Crude*.

b *Adjusted for age and sex*.

c *Adjusted for Model 2 + occupation*.

d *Adjusted for Model 3 + education*.

* *p < 0.05*.

Table 3 shows the main and interaction effects of psychosocial work environments and ADHD symptoms on work engagement among all participants. The interaction effects of psychosocial work environments × ASRS on work engagement were not significant.

**Table 3:** Main effects and interaction effects of ASRS and job control, job demands, and social support on work engagement among all participants.

	**Model 1^a^**	**Model 2^b^**	**Model 3^c^**	**Model 4^d^**	
	**β**	**β**	**β**	**β**	***P-value***
ASRS	−0.198^*^	−0.175^*^	−0.169^*^	−0.168^*^	< 0.01^*^
**Psychosocial work environments**					
Job control	0.216^*^	0.213^*^	0.206^*^	0.207^*^	< 0.01^*^
Job demands	0.249^*^	0.239^*^	0.230^*^	0.228^*^	< 0.01^*^
Social support	0.261^*^	0.265^*^	0.259^*^	0.258^*^	< 0.01^*^
**Psychosocial work environments** **×** **ASRS**					
Job control × ASRS	−0.029	−0.023	−0.021	−0.020	0.25
Job demands × ASRS	−0.023	−0.025	−0.024	−0.024	0.14
Social support × ASRS	−0.004	0.000	−0.001	−0.001	0.96
*R^2^*	0.308	0.327	0.332	0.333	
Adjusted *R*^2^	0.306	0.325	0.329	0.329	
Δ*R*^2^	0.308^*^	0.019^*^	0.006^*^	0.001	
Durbin–Watson				1.992	

*Work engagement was evaluated by Utrecht Work Engagement Scale*.

*β: Standardized coefficient (β)*.

a *Crude*.

b *Adjusted for age and sex*.

c *Adjusted for Model 2 + occupation*.

d *Adjusted for Model 3 + education*.

* *p < 0.05*.

For residual analyses, the Durbin-Watson statistic ranged from 1.988 to 2.041 (i.e., very near to the optimum of 2.0), which indicated there was almost no autocorrelation in the residuals (see Tables 2, 3).

## Discussion

This study demonstrated that there was a significant interaction effect of job control × ADHD symptoms and social support × ADHD symptoms on psychological distress. This study also found there was no interaction effect of psychosocial work environments × ADHD symptoms on work engagement.

## Psychosocial Work Environments and Psychological Distress

Multiple regression analysis showed an interaction effect of job control × ASRS severity on psychological distress. Greater job control was associated with lower psychological distress, and ADHD symptoms strengthened the association. These findings suggest that greater levels of job control are important to maintain mental health for workers with ADHD symptoms. However, past studies have shown that in workers with Autism Spectrum Disorder (ASD), greater job control is associated with poor health quality (35). ADHD and ASD are frequently comorbid, however the results of the past study may not apply to workers with an ADHD tendency who have comorbid ASD. Further research is needed to clarify the differences in psychosocial factors between individuals with ADHD and ASD and those with ADHD alone.

Multiple regression analysis showed no interaction effect of job demands × ASRS severity on psychological distress. Workers with ADHD often have the entrepreneurship and professionalism (27). These characteristics are fit to work with broad discretion, while these characteristics spontaneously encourage to concentrate on a task and then these way of working often lead to excessive workload. High job demands lead to poor health of workers with ADHD symptoms as is the case with workers without ADHD, and supervisors and occupational health staff pay attention to such circumstances. We observed that multiple regression analysis showed a significant interaction effect of social support × ADHD symptoms on psychological distress. Lack of social support might enhance the difficulty in work especially among workers with ADHD symptoms. These observations may support the importance of care by supervisors and occupational health staff for workers with ADHD.

## Psychosocial Work Environments and Work Engagement

We found that there was no significant interaction effect of job demands × ADHD symptoms and job control × ADHD symptoms on work engagement. Workers who interpret high job demands and high job control as a challenge may show improved work engagement. Although motivational themes identified by individuals with ADHD symptoms are similar to those identified by workers without ADHD symptoms, past research has shown that the details of the motivational factors differ, and that individuals with ADHD symptoms do not prefer predictable and familiar tasks (36). It may be necessary to devise strategies such as providing them with novel and diverse tasks. Further research is needed to reveal how psychosocial work environments are associated with work engagement for workers with ADHD symptoms.

Multiple regression analysis also did not show a significant interaction effect of social support × ADHD symptoms on work engagement. Bozionelos reported that incremental feedback on individual tasks resulted in greater improvements in work efficiency in workers with ADHD than in those without ADHD (28). It is difficult to explain this inconsistency, and we have to probe deeply how to perceive the support from supervisors or co-workers among workers with ADHD symptoms.

## Generalization of This Study Sample

The prevalence of workers with ADHD symptoms in this study was 5.6%, a slightly higher than the prevalence rate by structured interview [3.5% (5) and 4.2% (6)]. The past study reported that the prevalence rate by a self-administrated questionnaire cover a wide range [1.9% (7) and 6.0% (8)], while the participation rate of the former study was substantially low (35–38%) (7). The prevalence rate by a self-administrated questionnaire tend to be higher than that by structured interview, therefore the prevalence rate of workers with ADHD in this study was rarely difference in the past studies. Between the prevalence rate of men and women, there was no difference in this study. This finding contradicts some past reports, which showed that men had a higher prevalence rate of ADHD as diagnosed by structured interview (5, 6). In this study, we used a self-administrated questionnaire to identify ADHD symptoms. Women tend to internalize problems to a greater degree than men, which may lead to overestimation of ADHD and produce a high prevalence rate of ADHD among women when self-administered questionnaires are used. In similar studies using self-administered questionnaires, the prevalence rate of ADHD is either not different between males and females, or higher in females (7, 8).

The mental health status of workers with ADHD symptoms was poorer than that of workers without ADHD symptoms, which supports past findings (37). Among workers with ADHD symptoms, 7.9% of individuals scored higher than 13 points on the K6, which was the cut-off point for severe mental illness (38), compared with only 1.8% of individuals among workers without ADHD symptoms. This suggests that individuals with ADHD symptoms require medical treatment, and that occupational health staff needs to assist these individuals in obtaining psychiatric help.

Several limitations of our study warrant mention. First, all participants were from a single pharmaceutical company, and work engagement levels were higher than among other Japanese workers (39). This may be because the company emphasizes employee health and a pleasant working environment. Therefore, there may be limitations on the generalization of the study findings. Second, we did not collect information about marital status, whereas past studies using the K6 score or work engagement as a dependent variable adjusted for marital status. Third, this was a cross-sectional study that used a self-administered questionnaire, and no causal relationships between the variables can therefore be inferred. Further research is necessary to clarify the nature of these associations.

Despite these limitations, this study is to our knowledge the first to investigate the role of ADHD symptoms on the relationship between psychosocial work environments and negative/positive health. This work may serve as a basis for future discussion of the preferred psychosocial work environments for workers with ADHD symptoms.

## Author Contributions

MN, TN, and KM conceived and coordinated the project. MN, TN, and AI completed the analysis. MN and TN drafted the initial manuscript. MN, TN, AI, KM, and SM revised the manuscript, and commented on drafts of the report.

### Conflict of Interest Statement

The authors declare that the research was conducted in the absence of any commercial or financial relationships that could be construed as a potential conflict of interest.
